# Augmenting Qualitative Text Analysis with Natural Language Processing: Methodological Study

**DOI:** 10.2196/jmir.9702

**Published:** 2018-06-29

**Authors:** Timothy C Guetterman, Tammy Chang, Melissa DeJonckheere, Tanmay Basu, Elizabeth Scruggs, VG Vinod Vydiswaran

**Affiliations:** ^1^ Department of Family Medicine University of Michigan Ann Arbor, MI United States; ^2^ Institute for Healthcare Policy and Innovation University of Michigan Ann Arbor, MI United States; ^3^ Ramakrishna Mission Vivekananda Educational and Research Institute Belur Math, West Bengal India; ^4^ Department of Internal Medicine-Pediatrics University of Michigan Ann Arbor, MI United States; ^5^ Department of Learning Health Sciences Medical School University of Michigan Ann Arbor, MI United States; ^6^ School of Information University of Michigan Ann Arbor, MI United States

**Keywords:** qualitative research, natural language processing, text data, methodology, coding

## Abstract

**Background:**

Qualitative research methods are increasingly being used across disciplines because of their ability to help investigators understand the perspectives of participants in their own words. However, qualitative analysis is a laborious and resource-intensive process. To achieve depth, researchers are limited to smaller sample sizes when analyzing text data. One potential method to address this concern is natural language processing (NLP). Qualitative text analysis involves researchers reading data, assigning code labels, and iteratively developing findings; NLP has the potential to automate part of this process. Unfortunately, little methodological research has been done to compare automatic coding using NLP techniques and qualitative coding, which is critical to establish the viability of NLP as a useful, rigorous analysis procedure.

**Objective:**

The purpose of this study was to compare the utility of a traditional qualitative text analysis, an NLP analysis, and an augmented approach that combines qualitative and NLP methods.

**Methods:**

We conducted a 2-arm cross-over experiment to compare qualitative and NLP approaches to analyze data generated through 2 text (short message service) message survey questions, one about prescription drugs and the other about police interactions, sent to youth aged 14-24 years. We randomly assigned a question to each of the 2 experienced qualitative analysis teams for independent coding and analysis before receiving NLP results. A third team separately conducted NLP analysis of the same 2 questions. We examined the results of our analyses to compare (1) the similarity of findings derived, (2) the quality of inferences generated, and (3) the time spent in analysis.

**Results:**

The qualitative-only analysis for the drug question (n=58) yielded 4 major findings, whereas the NLP analysis yielded 3 findings that missed contextual elements. The qualitative and NLP-augmented analysis was the most comprehensive. For the police question (n=68), the qualitative-only analysis yielded 4 primary findings and the NLP-only analysis yielded 4 slightly different findings. Again, the augmented qualitative and NLP analysis was the most comprehensive and produced the highest quality inferences, increasing our depth of understanding (ie, details and frequencies). In terms of time, the NLP-only approach was quicker than the qualitative-only approach for the drug (120 vs 270 minutes) and police (40 vs 270 minutes) questions. An approach beginning with qualitative analysis followed by qualitative- or NLP-augmented analysis took longer time than that beginning with NLP for both drug (450 vs 240 minutes) and police (390 vs 220 minutes) questions.

**Conclusions:**

NLP provides both a foundation to code qualitatively more quickly and a method to validate qualitative findings. NLP methods were able to identify major themes found with traditional qualitative analysis but were not useful in identifying nuances. Traditional qualitative text analysis added important details and context.

## Introduction

### Background

Qualitative research methods are increasingly being used in social and health-related research because of their ability to help investigators understand nuances, contexts, and the perspectives of participants in their own words. Qualitative data can include images and videos, but text-based data is the most prevalent. The usual sources of text-based data are open-ended survey items, interview or focus group transcripts, and health record documents. However, text-based data needs different approaches for analysis compared with quantitative data to be able to answer complex research questions.

Qualitative text analysis is a process of analyzing qualitative text data, such as open-ended survey responses and interview transcripts. The process generally involves reading the data, assigning qualitative codes as succinct descriptors of meaning to text segments [1-3], and identifying themes that capture the major inferences to address study aims or research questions. Although the demand for qualitative research is high, it is a relatively labor-intensive process as researchers seek an in-depth understanding. Specifically, text data are dense, and researchers often underestimate the amount of data gathered through qualitative methods [3]. For instance, a 30-min interview yields about 10 pages of single-spaced transcribed text.

The overarching goal of qualitative research is often to provide an in-depth and nuanced report, typically by writing themes and a rich, thick description [3,4] that conveys the findings vividly and contextualizes them. To achieve depth, qualitative sample sizes tend to be small to allow researchers to complete the analysis and gain a detailed understanding. Despite the small sample size, the coding process alone takes considerable time due to the need to read all data, consider meaningful codes, assign relevant codes to segments, and discuss and reach agreement with other analysts. When sample sizes are large, a similar analysis of the entire database may become prohibitive in terms of time and effort, leading researchers to focus on a smaller, purposive subsample. However, what if it were possible to analyze larger qualitative databases with sufficient depth while reducing these barriers? Such analysis could leverage the depth of qualitative data with the generalizability of a larger, probabilistic sample.

One potential solution to mitigate the resource constraints of qualitative analysis is natural language processing (NLP). NLP is an area of research and application that explores how computers and automated algorithms can be used to understand and manipulate natural language text to accomplish useful, meaningful tasks [5]. It allows for the analysis of substantially larger text databases compared with the typical qualitative analysis methods and has been applied to data from electronic health records [6-8], PubMed [9], social media data [10], and text messages (short message service, SMS) [11]. However, research examining the methodological merits of NLP techniques is necessary to further consider NLP as a feasible and high-quality approach for qualitative analysis. Crowston et al [12] reported a case study about the use of NLP for qualitative analysis of messages to understand interactions between software teams. They found that NLP methods performed well in terms of an accurate number of codes identified and a reduction in the amount of text that humans would also have to code; they also increased the speed of coding. Aside from the groundbreaking study of Crowston et al [12], few methodological researchers have examined NLP through the viewpoint of qualitative analysis. Furthermore, we are aware of no other research that has directly compared qualitative analysis with those augmented with automated NLP approaches for text analysis. We conducted a study to compare NLP and qualitative text analysis on the basis of resources used, similarity of findings derived, and the quality of inferences generated. In this study, we applied both NLP and qualitative text analysis methods to a database of short open-ended survey responses from youth gathered via SMS text messages.

### Qualitative and Natural Language Processing Analysis of Text Data

Our intention is to in part bridge the gap between qualitative text analysis and NLP analysis by discussing the merits and limitations of the two approaches and how they complement each other. To understand our methods of text analysis more completely, we have provided an overview of qualitative text analysis and NLP approaches. Here, we have discussed our detailed methods of analysis of text data to provide background explanation because in the method section, we focus on how we compared these two approaches. The following section illuminates the two approaches for those who may be unfamiliar with either. As NLP relies on a computer algorithm to categorize free text, we offer substantially more details about how it is performed to make the use of a computer to analyze text more accessible.

### Qualitative Text Analysis

Although numerous methods for qualitative text analysis exist, the general approach involves reading the data and assigning codes to text segments [2]. A code is a concise qualitative label (eg, “exercise,” “staying healthy,” and “harmful”) to identify the meaning in a segment of text. For example, “I like to exercise” might be coded by investigators as “exercise.” Applying the qualitative thematic text analysis approach of Kuckartz [2], investigators identify codes through the analysis based on reading the data. The process of assigning a code label is subjective; however, procedures are available to establish intercoder agreement through a consensus process. For example, 2 or more researchers may code the same text initially and discuss discrepancies, such as using different codes or synonyms that have virtually the same meaning for a code label. In addition, researchers might use sensitizing concepts [13] to identify some codes a priori based on a theoretical or a conceptual model guiding the research. Considerable debate prevails around that practice, which qualitative purists might consider as too postpositivistic or deductive [1].

Analysis continues as researchers refine the codes used, define each code more precisely, and iteratively develop the code book. Researchers then identify themes or categories that represent major findings of the analysis [2]. Identifying themes is a process of examining patterns and similarities among codes and then interrelating the themes. The themes then comprise the major findings of the analysis. In this study, we applied this thematic text analysis approach to analyze the text data gathered from youth.

### Natural Language Processing Framework

NLP is a subfield of computer science and linguistics that deals with algorithms, methodologies, and tools to analyze natural language text and studies grammatical, syntactic, and semantic structure of text. In our study, for example, the SMS text messages comprised open-ended short responses by participants of the survey. Depending on the type of questions, the text responses could be a simple “next,” a preferred choice (eg, “yes” or “no”), or a more verbose answer, for example, in response to a “Why?” question. The preferred choice responses were cleaned by removing punctuations and capitalization and normalized by correcting spelling errors. The “Why?” question responses were also processed with these data cleaning steps, after which they were clustered based on similarity of words.

Word similarity can be computed based on the relative distance between words in a hierarchical word ontology known as WordNet. WordNet is a database for the English language that contains words and multiword phrases and organizes nouns, verbs, adjectives, and adverbs into more than 117,000 synonym sets (known as synsets) [14]. A synset is a set of words that have the same meaning (cognitive synonyms). The synsets are further organized into a hierarchy of “is-a” relations (hypernyms and hyponyms), which could be used to compute similarity of a pair of words. For example, the words “exercise” and “workout” are synonyms and “stretching,” “yoga,” and “calisthenics” are hyponyms of “exercise,” whereas “exertion” is the hypernym of “exercise.” Nouns indicating other exertion activities such as “straining” and “pull” are siblings of “exercise” in the WordNet hierarchy (ie, “straining,” “pull,” and “exercise” are all hyponyms of “exertion”). Based on edge distance between appropriate synsets in this tree-like structure, one could consider that exercise and workout are very similar (an edge distance of 0), exercise and yoga are quite similar (an edge distance of 1), whereas exercise and straining are even less similar (an edge distance of 2). In contrast, the words exercise and weight, although they appear to be related, have a large tree edge distance and are considered not similar—while one is an activity, the other is an artifact. The performance of WordNet to measure the semantic orientations of adjectives has been measured against human judgment and was found to be very effective [15].

Several similarity measures have been proposed that use the edge distance and other factors in computing word-to-word similarity [16,17]. For example, Wei et al [18] used WordNet to find semantic similarity between words instead of content similarity to improve the effectiveness of automatic text clustering. They explored several semantic similarity measures over WordNet, for example, Leacock and Chodorow similarity [19] and Wu-Palmer similarity [20] for finding semantic similarity in the text clustering technique. They found that Wu-Palmer similarity was a better measure to capture the semantic similarity between words in a text clustering application. Wu-Palmer similarity between two words is the path length to the root node from the least common subsumer (LCS) of the two words in WordNet [20]. LCS is the most specific concept a pair of words share as an ancestor. For example, in the sample WordNet hierarchy given above, the LCS for “yoga” and “pull” is “exercise,” since it is the most specific ancestor of “yoga” and “pull.” In the Wu-Palmer similarity measure, the path length is normalized by dividing the sum of the path lengths from the individual words to the root word [20]. The Wu-Palmer similarity has been used to find semantic relatedness between concepts in many genres of text, including biomedical texts [17,21,22].

In this study, we used the Wu-Palmer similarity measure [23] to find semantic similarity between words. Researchers use the word similarity measures to identify clusters or concepts based on synonyms and very similar word pairs (eg, Wu-Palmer similarity >0.9). Assigning similar words and phrases to one concept group is similar to using a code label in qualitative text analysis. However, through the use of NLP, this process is automated. The detailed description of the proposed NLP framework to find different clusters of similar words from the short messages is as follows:

1. All nouns and pronouns were extracted from the short message texts and converted to lower case. We did not consider the other parts of speech because they were found to have little contribution toward identifying concepts from the SMS text messages. The same was observed empirically.
2. A vocabulary was created with unique nouns and adjectives (ie, multiple occurrences of a word are discarded).
3. Synsets of each word in the vocabulary are generated using WordNet. Note that a word may have more than one synset as described earlier.
4. A pair of words were grouped together if the Wu-Palmer similarity between any pair of synsets, one generated from the first word and the other from the second word, is >0.9. It may be noted that the Wu-Palmer similarity score ranges from 0 to 1, both inclusive, and 1 indicates the highest similarity. This step was repeated for all pairs of words in the vocabulary.
5. Derivationally related forms of each word in the word pairs were generated, for example, “honest” is derivationally related to “honesty” as generated by WordNet.
6. The most similar pair of words and their derivational forms were combined to create a cluster. This led to several word clusters to be created from the vocabulary.
7. A pair of clusters was merged, if they had at least 50% common members (ie, words). The process continued until no more merges could take place. The method terminated automatically upon satisfying the given condition and generated the final clusters of words. These clusters indicated different senses and semantic meanings present in the given database of SMS text messages.

### Study Background and Context

To conduct this research, we used SMS text message survey data gathered from the MyVoice study [11]. MyVoice is a national SMS text message poll of youth, in which 3-5 SMS text message questions were sent to a national sample of participants aged 14-24 years on a weekly basis. Questions were typically open-ended, and topics range from current events to specific health concerns. The purpose of this study was to describe the merits of integrating NLP and manual qualitative data analysis based on our direct comparison of the 2 approaches applied to a dataset. Our goal was not to prefer one approach over the other but to critically reflect on the value and limitations of each approach alone in addition to the integration of the two. This article provides guidance to researchers in deciding to use NLP techniques and manual qualitative text analysis.

## Methods

### Overview

To compare NLP methods and qualitative analysis, we conducted a modified 2-arm cross-over experiment. Our primary outcomes were comparing the similarity of thematic findings and time taken to analyze based on person hours. We were also particularly interested in documenting the process for each arm. A different, experienced coding team conducted the analysis for each approach. Two qualitative analysis teams (each with 2 of the authors, MD, TC, ES, and TG) independently coded and generated findings from two different datasets, one focused on opinions about prescription drug use and the other focused on interactions with police. Our analysis followed the qualitative text analysis process as noted previously. The 2 teams used MAXQDA 12 qualitative software (VERBI GmbH; Berlin, Germany) to facilitate the analysis process. We randomly assigned the datasets to each team to begin the analysis. Simultaneously, a third team (VV and TB) independently conducted NLP analysis with each dataset. We computed word similarity over all pairs of nouns in responses to the 2 survey questions. Using the criterion of Wu-Palmer similarity >0.9, we then grouped survey responses that contained synonyms and very similar word pairs into a concept group.

The qualitative analysis teams received the NLP results for the dataset they had already coded to augment their analysis and reconsider their conclusions. The cross-over then occurred as the 2 qualitative teams received the NLP data *first* from the other dataset they had no prior exposure to. The reason for having a cross-over design was to account for team effects and different question set effects. The teams proceeded with qualitative analysis after reviewing the NLP results and then developed conclusions. In summary, we had the following 2 cross-team comparison conditions: (1) NLP-only followed by augmented qualitative or NLP analysis and (2) qualitative analysis-only followed by an augmented qualitative or NLP analysis.

### Data Sources

We used a subset of open-ended text data identified from a larger project and applied each of the 2 coding approaches. Open-ended questions enabled the participants to construct a response without being constrained by response options and allowed them to explain their response. The data consisted of responses to an SMS text message-based survey with young adults aged 14-24 years (mean 18 years). We focused on 2 questions, one about drugs and the other about experiences with police, respectively. The drug question was “What do you think is more dangerous: taking a prescription drug that is not yours or an illegal drug? Why?” The police question was “What have your experiences with police been like?”

### Analysis

To address our methodological purpose, we carefully examined the results of our data analysis. Our goals for this methodological analysis were to understand (1) how the thematic findings differ when beginning with qualitative-only coding followed by the qualitative/NLP augmented coding compared with beginning with NLP followed by NLP/qualitative augmented coding; (2) how the process led to additions, deletions, changes in codes or changes in the definitions of codes; and (3) how the conclusions derived differ between the 2 teams. We also calculated the percent agreement between the 2 researchers in each team in coding of the same dataset to check for consistency. We found acceptable agreement [1] with 62 of 88 (70%) coded segment agreements and 72 of 84 (87%) agreements, respectively, for the 2 teams. More importantly, each team engaged in the process of discussing codes line-by-line and reconciling differences through consensus. In addition, the teams recorded process notes, including the time spent in each analysis. Finally, we compared the inferences generated by each standalone approach and a qualitative/NLP augmented approach.

## Results

### Overview

We have briefly summarized the findings of our analysis of text data using the following different approaches: qualitative coding, qualitative followed by NLP-augmented coding, NLP-only coding, and NLP followed by qualitative-augmented coding. The findings reported in tables are summaries from the teams after their independent analysis and before any discussion between teams. After reviewing the coding and conclusions, we have discussed the results of our methodological analysis of this process.

### Comparing Qualitative, Natural Language Processing, and Augmented Coding Approaches for Text Analysis

In total, 84 individuals answered at least one of the 2 sets of questions; 58 answered the drug question and 68 answered the police question, which were the focus of our analysis. The demographic questions were not required, and demographics were available for 66 of the 84 individuals (Table 1).

#### Drug Question (What do You Think is More Dangerous: Taking a Prescription Drug That is Not Yours or an Illegal Drug? Why?)

The thematic findings of the 2 teams were relatively consistent (Tables 2 and 3). The qualitative-only analysis for this question yielded 4 major findings. The perspectives of youth respondents included that prescription and illegal drugs are equally dangerous and that the degree of danger depends on the situation. Other youth explained that either drug could be more dangerous, depending on the intention of use and whether the drug was prescribed. The NLP-only findings were somewhat similar but also driven by word frequencies. The major NLP-only findings were that (1) youth were divided as to which was more dangerous, (2) more noted that illegal drugs were more dangerous, but (3) 11 youth responded that the danger depends on the situation. However, the context was missing from the NLP-only analysis. For example, some wrote about side effects and harm to the body, but we could not determine from the NLP results whether these comments referred to illegal drugs or prescription drugs that are not theirs. Finally, the qualitative followed by NLP-augmented results were more comprehensive. For example, a qualitative-only thematic finding was that either prescription or illegal drugs could be more dangerous and stigma was an issue. However, when we examined the NLP-generated data, we recognized that “stigma” was incomplete and actually more complex. We added that stigma was related to the discreetness of obtaining prescription drugs compared with buying street drugs.

The 2 teams—one began with qualitative-only followed by NLP-augmented coding and the other began with NLP-only followed by qualitative-augmented coding—reached very similar conclusions. From the augmented analysis, both teams added to findings about the legality of the drug, whether the ingredients are known, and Food and Drug Administration approval. For one team, these new points rose to the level of major conclusions, while the other tended to add them as details, perhaps reflecting more of a stylistic difference between teams. However, for both teams, the legality issue was clearly a major thematic finding that the augmented qualitative and NLP approach added.

#### Police Question (What Have Your Experiences With Police Been Like?)

Tables 4 and 5 compare thematic findings for the 2 teams and different approaches to coding the police data. Again, findings were relatively consistent. The qualitative-only results for this question yielded the following 4 primary findings: (1) about one-third of youth had no real interaction with police to comment on, (2) the majority who had interaction had a positive experience, (3) some noted concerns about racism, and (4) a small group described the importance of police to maintain public safety. The NLP-only analysis yielded the following 4 slightly different findings: (1) some had few experiences, (2) several youth had positive experiences, though some were “bad,” (3) individual relationships and characteristics affected experiences, and (4) youth could point to specific “situations” with police.

As with the drug question, the qualitative- and NLP-augmented results for the police question were the most comprehensive. Based on the additional review of the NLP police data, “good/positive” and “bad” commentary were most frequent, which reinforces the conclusion from the qualitative-only phase. The NLP data did reflect several occasional references to race (white) or gender (women). Furthermore, multiple mentions of police force as respectful and assurance of security lend credence to the qualitative conclusion that police are needed to maintain public safety. In general, the NLP data were unable to pick up on nuance (eg, that some individuals described both positive and negative experiences at the same time) but ultimately reinforced the basic conclusions noted by the qualitative team. Furthermore, NLP data revealed that gender, in addition to race, was related to participant’s experiences and feelings surrounding the police. In brief, the augmented NLP and qualitative analysis increased the depth of understanding (ie, details and frequencies), but the overall findings were quite similar.

### Methodological Comparison of Methods

Our analysis revealed 3 methodological insights related to the process of using NLP data, how NLP and qualitative-augmented analysis tended to yield more information, and observations about how the ordering of analysis affected the process. First, we developed a process to use and review NLP data, which was somewhat different from our qualitative coding process. In using NLP, we first reviewed the entire file sent by the NLP team. It consisted of a spreadsheet of words, synonyms in the dataset, and relative frequencies (see Table 6 for an example of NLP output).

We looked for response phrases that simply repeated words in the question, typically listed with high frequency in the list, and discounted those. We reviewed all words and examples, highlighting those that stood out. For analyses that began with qualitative coding, followed by NLP, we were particularly keen to find NLP concepts that we missed qualitatively. At this point, it was helpful to create a conceptual model by drawing a map relating codes. If we had findings from an initial qualitative analysis, we then integrated NLP data with what we knew from the qualitative findings. Finally, we noted a potential difference in achieving data saturation between NLP and qualitative analysis. Data saturation is the point at which themes are sufficiently complex and collecting additional data is not adding to findings [24]. In NLP analysis, the point of saturation tended to occur while we reviewed more frequent words and before we reached less frequent words. In NLP, saturation seemed to be dependent on the frequency of ideas rather than the complexity of themes. In contrast, in qualitative coding, we reached saturation at some point while reading through the data. We noted that with qualitative coding, saturation was more dependent on the order in which text responses appeared throughout this study, and we continued to analyze all responses.

The different approaches yielded similar thematic findings, but the NLP- or qualitative-augmented coding produced more information than the qualitative-only or NLP-only approaches. 

**Table 1:** Participant demographic information.

Variable		Drug response (n=48)	Police response (n=59)	Drug or police response (n=66)
Age, mean (SD)		18.5 (2.2)	18.3 (2.5)	18.3 (2.4)
**Gender, n (%)**				
	Female	28 (58.3)	33 (55.9)	37 (56.1)
	Male	18 (37.5)	25 (42.4)	27 (40.9)
	Other	2 (4.2)	1 (1.7)	2 (3.0)
**Race, n (%)**				
	White	26 (54.2)	36 (61.0)	38 (57.6)
	Black	8 (16.7)	9 (15.3)	11 (16.7)
	Asian	7 (14.6)	7 (11.9)	8 (12.1)
	Other (including multiracial)	7 (14.6)	7 (11.9)	9 (13.6)
	Hispanic^a^	0 (0.0)	3 (6.7)	3 (6.4)
**Education, n (%)**				
	<High school	19 (39.6)	28 (47.5)	31 (47.0)
	High school grade	7 (14.6)	5 (8.5)	7 (10.6)
	Some college	17 (35.4)	19 (32.2)	20 (30.3)
	College grade (BA+)	5 (10.4)	7 (11.9)	8 (12.1)
**Parent education^a^, n (%)**				
	High school or less	1 (3.5)	2 (4.4)	2 (4.3)
	Some college or 2-year degree	4 (13.8)	5 (11.1)	5 (10.6)
	BA but less than Masters	7 (24.1)	8 (17.8)	9 (19.2)
	Masters but less than PhD	9 (31.0)	18 (40.0)	19 (40.4)
	PhD	8 (27.6)	12 (26.7)	12 (25.5)
**Primarily living with^a^, n (%)**				
	Parents	22 (75.9)	33 (73.3)	35 (74.5)
	Dorm	0 (0.0)	1 (2.2)	1 (2.1)
	Sharing an apartment with other people	7 (24.1)	8 (17.8)	8 (17.0)
	Other	0 (0.0)	3 (6.7)	3 (6.4)
**Family size^a^, n (%)**				
	1-3	6 (20.7)	9(20.0)	10 (21.3)
	4-6	17 (58.6)	29 (64.4)	29 (61.7)
	7-10	5 (17.2)	7 (15.6)	7 (14.9)
	11+	1 (3.5)	0 (0.0)	1 (2.1)
**Parent’s marital status^a^, n (%)**				
	Married or together	21 (72.4)	36 (80.0)	36 (76.6)
	Divorced or separated	7 (24.1)	7 (15.6)	9 (19.2)
	Other (widowed, unsure)	1 (3.5)	2 (4.4)	2 (4.3)

^a^Sample sizes are as follows: Drug response (n=29); police response (n=45); and drug or police response (n=47). Participants were not required to provide demographic information, so the n for respective demographic questions in this table is lower than the total number of participants. Because some responded to both questions, we have 3 columns of demographic information. There are fewer responses for ethnicity, parent’s education, primary living situation, family size, and parent’s marital status due to those questions not being asked to the subset of individuals who had demographics requested twice. The third column displays data for those who responded to at least one question.

**Table 2:** Comparison of findings derived from qualitative-only and qualitative followed by natural language processing-augmented approaches to coding for the drug question (n=58). Key aspects of each finding are italicized.

Theme	Qualitative only^a^	Qualitative (natural language processing augmented)^b^
Prescription drugs and illegal drugs	Prescription drugs and illegal drugs are *equally dangerous* because both are serious, could harm you, and are illegal.	Prescription drugs and illegal drugs are *equally dangerous* because both are serious, could harm you, and are illegal.
Danger	*Danger depends* on the situation, the amount of drug taken, type of drug, whether or not it was prescribed to you. Distinction between a medical danger versus a legal danger.	*Danger depends* on the situation, the amount of drug taken, type of drug, whether or not it was prescribed to you. Distinction between a medical danger versus a legal danger.
*Respondent chose either/or*	Either Rx^c^ drugs or illegal drugs could be more dangerous based on addictiveness, accessibility, prevalence, overdose, or danger. Side effects: known or unknown. Stigma.	Either Rx drugs or illegal drugs could be more dangerous based on addictiveness, accessibility, prevalence, overdose, or danger. Side effects: known or unknown. Stigma of getting drugs off the street versus discreetness of “popping” Rx pills.
*Intention or appropriateness*	Is the drug safe for everyone or unsafe for some people depending on whether prescription was prescribed to you.	Is the drug safe for everyone or unsafe for some people depending on whether prescription was prescribed to you.
*Ingredients*	—	What the drugs consisted of. Mixing Rx versus unknown contents of street drugs versus taking Rx you do not know what they are.
Legality	—	Legal more prominent; the *legality of using other prescriptions and the legality of street drugs* and what that meant about street drug safety or regulations.
*Government involvement*	—	*Food and Drug Administration approval* and regulations versus “illegal” for a reason.
*Harm to body*	—	Mortality was often mentioned (overdose, “something that could kill you”).
*Specific drugs* mentioned	—	“Weed, heroin, cocaine, meth, alcohol, smoking” and using them for comparisons for safety and side effects or addictiveness.

^a^Time required (person min): 270.

^b^Time required (person min): 180.

^c^Rx: prescription medication.

**Table 3:** Comparison of findings derived from natural language processing–only and natural language processing followed by qualitative-augmented approaches to coding for the drug question (n=58). Key aspects of each finding are italicized.

Theme	Natural language processing only^a^	Natural language processing (qualitative augmented)^a^
Prescription drugs and illegal drugs	Respondents seemed *divided* between whether illegal drugs or medicines were more dangerous.	Of the 58 respondents, 24 noted that illegal *drugs were more dangerous*, 15 thought both were *equally* dangerous, and 11 answered *prescription drugs*. 10 argued that it depended on the context.
Danger	11 respondents noted that it *depends* or similar as to what is more dangerous. Some noted it depends on the reason for using either.	For some, the question of danger *depends* on who owns the drug, the situation, the type of drug, and the ease of access. Several felt that the answer depended on what type of drug (either illegal or prescription), how much, and for what. A few felt that certain illegal drugs (eg, marijuana) were less dangerous than legal drugs (eg, alcohol).
*Government involvement*	It seemed that more wrote that *illegal drugs were more dangerous.*	*Illegal drugs may be more dangerous* because they are not medically cleared, federally regulated, nor approved by the Food and Drug Administration. They may contain traces of other substances. Respondents felt uncertain about where the illegal drug might come from or what it really contained.
*Prescription drugs*	—	*Prescription drugs may be more dangerous* because they are easy to access and not illegal, so more people may feel comfortable taking them.
*Intention or appropriateness*	—	Several respondents noted that taking a prescription drug that is not yours is *illegal* too.
*Side effects and harm to the body*	Respondents wrote about *side effects* (n=5) and *harm to the body* (n=6), but we cannot determine whether it referred to prescription medicine or illegal drugs.	—

^a^Time required (person min): 120.

**Table 4:** Comparison of findings derived from qualitative-only and qualitative followed by natural language processing-augmented approaches to coding for the police question (n=68). Key aspects of each finding are italicized.

Theme	Qualitative only^a^	Qualitative (natural language processing augmented)^b^
Interactions	For those who had interaction, the majority of participants described *interactions as positive* (eg, “pleasant,” “positive,” “decent”). Others reported negative interactions (eg, “bad,” “not so good”). Some of the individuals who described positive experiences also gave negative experiences, such as describing a sense of fear (“Positive but I am always scared interacting with them”).	For those who had interaction, the majority of participants described *interactions as positive* (eg, “pleasant,” “positive,” “decent”). Others reported negative interactions (eg, “bad,” “not so good”). Few described the interactions as negative or bad. Some of the individuals who described positive experiences also gave negative experiences, such as describing a sense of fear (“Positive but I am always scared interacting with them”).
Racism/gender differences	Some individuals wrote about major concerns with *racism among police*.	Some individuals wrote about major concerns with *racism among police*. Several mentioned *gender differences* in how they were treated.
Public safety	A small group described police as *needed in order to maintain public safety*.	A small group described police as *needed in order to maintain public safety*.

^a^Time required (person min): 270.

^b^Time required (person min): 120.

**Table 5:** Comparison of findings derived from different approaches to coding for the police question (n=68). Key aspects of each finding are italicized.

Theme	Natural language processing only^a^	Natural language processing (qualitative augmented)^b^
Number of experiences	Some youth reported *few experiences* with police.	Some youth reported *few or no experiences* with police (n=17). Others reported “inconsequential” interaction (n=2).
Interaction	Many youths reported good or *positive experiences* with police. Other words in this theme include friendly, respectful, pleasant, and helpful. Others reported *“bad” experiences*, unpleasant, aggressive, and mean (less frequency words).	Many youths reported good or *positive experiences* with police (n=32). Other words in this theme include friendly, respectful, pleasant, and helpful. Others reported *“bad” experiences*, unpleasant, aggressive, mean (n=7). Some reported good and bad (n=2).
Situations	Youth can point to *“situations,” “moments,” and “times” when they interacted with police*. Some of these situations were at concerts, sporting events, or at their schools.	Youth can point to *“situations,” “moments,” and “times” that they interacted with police*. Some of these situations were at concerts, sporting events, or at their schools.
Avoidance	—	*Avoid situations with police*, makes people nervous, excessive force, seem mean, cause fear.
Individual characteristics	*Individual characteristics and relationships* come into play: white, woman, young, friends, or parents.	*Individual characteristics and relationships* come into play: white, woman, young, friends, or parents. Specifically, race (n=5) and gender (n=2).

^a^Time required (person min): 40.

^b^Time required (person min): 180.

**Table 6:** Example natural language processing output from the drug dataset.

Code word	Frequency	Similar words	Data segments
Medicines	98	Medication, medicine, medicate, prescription, drug	“an illegal drug” “true for specific drugs” “addictive than prescription medication” “whereas prescription medications are legal” “most dangerous drugs out there” “because prescription drugs are specifically”
Illegal	48	—	“is the illegal drug yours” “doing an illegal drug since” “compared to illegal drugs” “both are illegal in my”
Prescription	26	Prescriptions	“addictive than prescription medication” “some prescription medicines are” “least the prescription medication is” “very dangerous prescription medicines but”
Dangerous	14	—	“are equally dangerous” “is more dangerous because it” “is physically/mentally dangerous and illegal”
Depends	11	—	“it depends on which”
More	9	—	“can be more addictive than” “medicines are more dangerous than” “a lot more overdoses than” “drug is more dangerous than” “can be more powerful and”

**Figure 1:**
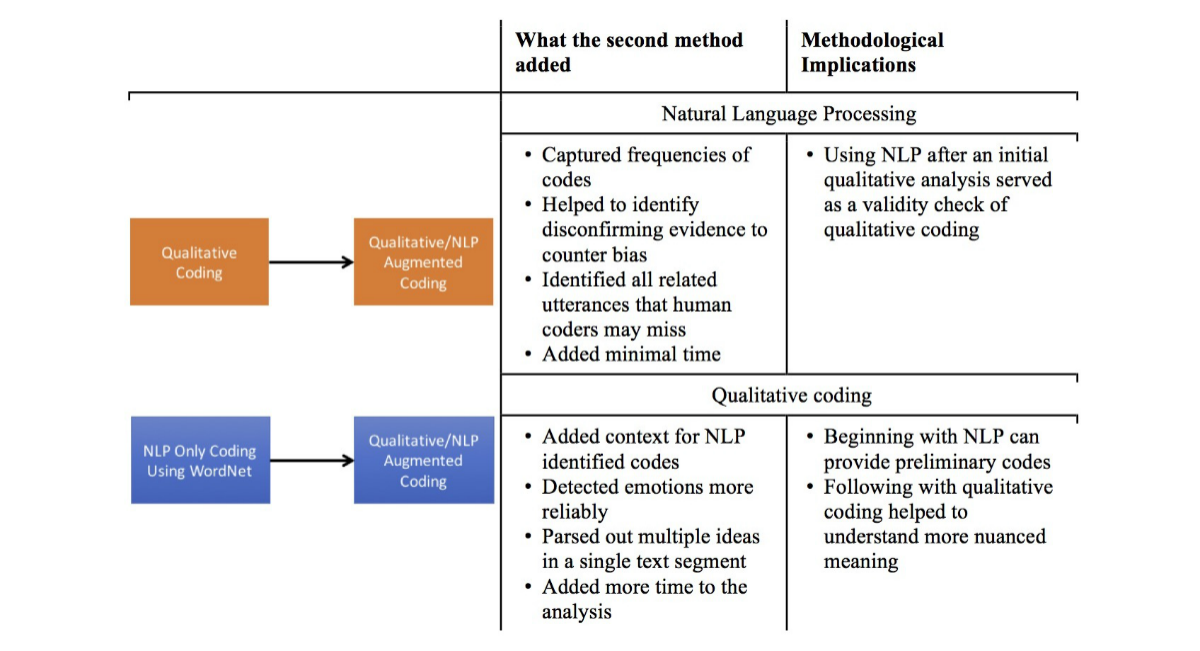
Summary of methodological findings. NLP: natural language processing.

Qualitative coding was beneficial in our analysis because it added critical contextual understanding that helped to interpret responses. For example, in the police data, the qualitative coding helped the analysts to identify emotions, such as fear, that were not evident in the NLP analysis. Qualitative coding also helped us to identify full quotes that we might include in a report of findings. On the other hand, NLP was beneficial in terms of concept and code frequencies. It can add a validity check, which is concerned with the accuracy of qualitative findings [3]. Although we were able to determine the relative frequency (eg, quantifying comments by “all,” “most,” “some,” “few,” etc) of codes through qualitative coding, the process of finding NLP word frequencies was more efficient. Therefore, the augmented approach of combining both NLP and qualitative approaches leveraged the strengths of each. The NLP- or qualitative-augmented coding led to more conclusions and more depth within conclusions.

Our process notes yielded further insight into the ordering of augmenting one method with another. As anticipated, the NLP-only approach was quickest at 120 minutes for the drug question and 40 minutes for the police question, whereas the qualitative-only approach was longest at 270 minutes for both questions. However, we found a difference, an approach beginning with qualitative analysis followed by qualitative- or NLP-augmented analysis took longer time than that beginning with NLP for both drug (450 vs 240 minutes) and police questions (390 vs 220 minutes). As noted in Figure 1, including the second method added different benefits depending on the order. Our subjective preference—from both teams—was to begin with qualitative coding and then augment it with NLP afterward, which was unexpected. Prior to the study, we anticipated that it would be more helpful to begin with NLP, but all 4 analysts felt that it was more difficult to start with NLP word frequencies because we were missing the context of the codes. On the other hand, beginning with qualitative coding, we gained a stronger sense of the data and found it helpful to add NLP as a validation strategy. Despite our subjective preference, we did not find substantive differences in the findings when beginning with one method versus the other.

## Discussion

### Principal Findings

In brief, our primary conclusion is that NLP is a viable method for coding text data, and it is particularly useful when augmenting manual qualitative coding. Each method alone yielded relatively consistent results, but the combination (ie, augmented) analysis clearly added more detail across the 2 questions, a finding consistent with the study of Crowston et al [12]. The final, augmented findings from the 2 teams were relatively similar, which suggests that we are not merely detecting differences among teams but also obtained insights into the ordering of the augmenting of approaches. We observed a similar pattern across questions, in which the augmented analysis yielded the most complete details. Finally, though we noted that we achieved data saturation with both NLP and qualitative approaches, saturation may be a nonissue for NLP of data gathered through probabilistic sampling to achieve generalization.

### Limitations

Several limitations of this study and the approach must be noted. First, this research was based on responses to 2 short open-ended survey questions. Our findings may not transfer to longer responses such as interview transcripts because NLP’s challenge with nuance and contextual responses might increase. Regarding time efficiency, the times we recorded were based on a relatively small text database of short snippets. Although the augmented approach took more time in our comparison, we anticipate that with larger datasets, the efficiency of an NLP-augmented approach will prevail and take less time. Further research is needed to compare the approaches with interviews and other text data. Second, it is possible that some of our findings actually reflect differences among the 2 qualitative teams. However, we attempted to counterbalance those concerns through the cross-over design. Third, each method has its own limitations. Qualitative coding generally eschews frequencies, whereas NLP privileges that concept. Thus, a natural question is whether the two are comparable. Furthermore, qualitative coding is limited to a purposeful, rather than a probabilistic sample that is generalizable. In addition, WordNet is carefully curated and does not reflect current events, slang, or nonsemantic use of terms. For this reason, it does have some limitations for analysis of adolescent language and SMS text message data. Other limitations are related to our particular simple NLP approach. Other more complex approaches, such as sentiment analysis, may capture the context better. A future study can directly compare NLP output with qualitative analysis. Nevertheless, we believe that the limitations of each approach add further credence to the idea of an augmented qualitative and NLP approach.

### NLP Coding and Context

It is important to note that NLP helped quantify, organize, and categorize responses quickly—providing an accurate overview of the major themes. NLP by itself can miss the context of what was being said, especially when emotion was involved (eg, “fear” of police), similar to the findings of Crowston et al [12]. We recommend a qualitative-augmented approach to understand the context. Practically, it might involve taking a subsample of a larger NLP analysis to augment with qualitative coding to understand the context.

Moreover, NLP has the potential to add value to manual qualitative analysis procedures. NLP does capture the relative frequency of particular codes and words, which can add important information about the weight of what was communicated to complement the context provided by qualitative methods. In general, NLP can provide a validity check of qualitative findings by providing a second method to triangulate findings or by helping the researcher to systematically search for disconfirming evidence. After establishing preliminary themes, qualitative researchers might use NLP to engage in this qualitative validation procedure to look for contrary data, exceptions, and alternate perspectives in examining whether evidence supports or disconfirms findings as reported by Creswell and Miller [25]. NLP offers the ability to search for all text and utterances that human readers may overlook. In addition to these potential benefits, using NLP itself adds minimal time.

### Nature of Data Analyzed

We noticed another contextual issue based on the nature of the question analyzed. The drug question required the participants to compare (ie, an illegal drug or a prescription drug that is not yours). NLP alone is not well equipped to understand the context of comments and which of the 2 types of drugs participants were commenting on. Alternatively, the question could have been separated into 2 items to facilitate an NLP-only analysis. The police dataset had similar nuances with added qualifiers provided by some. Therefore, we recommend any comparative questions be analyzed through an NLP and qualitative augmented approach.

Although our data were from a larger study of adolescent perspectives on policy and health topics, the methods we used are agnostic of the topic domain. The augmented approach can be applied to a wide variety of health, medical, and topics from other domains. Furthermore, it is applicable to other data sources, including traditional survey responses, social media snippets, or documents. Both the questions we analyzed consisted of relatively short text segments and similar sample sizes of 58 and 68, respectively. Although we found no time savings with the NLP and qualitative augmented approach, as the sample size increases, the efficiencies of NLP will likely be clearer. One way to achieve time efficiency is by applying NLP to the entire dataset and qualitatively coding a smaller subsample. The implication for future research and development is to advance how the method can be applied to longer text, such as qualitative semistructured interview transcripts and more complex responses rather than the domain of inquiry, as this technology should be applicable to any text data. Therefore, we urge further research applying an NLP and qualitative augmented approach to interview transcripts and other forms of text data.

### Conclusions

NLP methods were able to identify major themes found with traditional qualitative analysis, but the approach was not useful at identifying nuances. Subsequent traditional qualitative text analysis added important details and context. Researchers using NLP techniques might want to consider analyzing even a portion of data with qualitative text analysis to ensure that important context is not missed.

NLP provides both a foundation to code qualitatively more quickly and a method of validation. NLP can help researchers conduct qualitative analysis more quickly because a coding rubric may become apparent in the NLP output. Findings from NLP by itself may be appropriate for analysis that must be done rapidly for a focused question (ie, policy questions, formative program evaluation could improve processes in real-time, community needs assessments). Finally, NLP can add a validity check of qualitative findings by adding frequency counts and larger sample sizes to the conclusions drawn from qualitative analysis when needed to address the research questions.

## Abbreviations

NLP: natural language processing
LCS: least common subsumer
SMS: short message service
